# Cancer-Associated Retinopathy as the Sole Presenting Sign of Small Cell Lung Cancer: A Case Report

**DOI:** 10.7759/cureus.76759

**Published:** 2025-01-01

**Authors:** Quan H Phung, Collin McClelland, Hem Desai, Lynn Messersmith, Lucia Reinhardt, Robert Kratzke

**Affiliations:** 1 Medicine, Division of Hematology, Oncology, and Transplantation, University of Minnesota, Minneapolis, USA; 2 Ophthalmology and Visual Neurosciences, University of Minnesota, Minneapolis, USA; 3 Medicine, Division of Pulmonary, Allergy, Critical Care, and Sleep Medicine, University of Minnesota, Minneapolis, USA; 4 Laboratory Medicine and Pathology, University of Minnesota, Minneapolis, USA

**Keywords:** cancer-associated retinopathy, case report, paraneoplastic syndrome, small cell lung cancer, vision loss

## Abstract

Paraneoplastic syndromes are a rare group of disorders involving an immune reaction to cancer. Since paraneoplastic syndromes can impact any organ system, presenting symptoms can vary dramatically, which can make them difficult to detect and diagnose. Paraneoplastic visual symptoms are a rare and potentially early manifestation of an underlying malignancy. This case report describes a patient who presented with progressive, bilateral vision loss. Neuro-ophthalmology evaluation revealed severe bilateral outer retinal atrophy on optical coherence tomography and vitritis. This raised a concern for cancer-associated retinopathy, a visual paraneoplastic process. Further imaging revealed a spiculated lung mass and led to a diagnosis of small-cell lung cancer. Unfortunately, despite early initiation of corticosteroids and intravenous immunoglobulin, he did not have significant visual improvements. However, he did have a significant initial tumor response to carboplatin, etoposide, and atezolizumab. Ultimately, astute recognition of this paraneoplastic process led to an early diagnosis and initiation of treatment for small cell lung cancer. We describe this case with the goal of helping other providers consider visual paraneoplastic processes among their differential diagnoses, especially among patients with unusual presenting symptoms or who have underlying risk factors for malignancy.

## Introduction

Paraneoplastic syndromes are a heterogeneous group of clinical syndromes that are associated with certain cancers, such as lung, breast, gynecologic, and hematologic malignancies. These symptoms are not derived from the direct structural effect of primary tumors or metastases, but from peptides, hormones, or antibodies acting at sites remote from the neoplasm [1]. Paraneoplastic syndromes can impact a diverse set of organ systems with varied presentations, such as hyponatremia (due to syndrome of inappropriate anti-diuretic hormone), hypercalcemia, Cushing’s syndrome, and neuromuscular weakness. Paraneoplastic visual syndromes tend to result from immune cross-reactivity between tumor cells and the afferent visual pathways (e.g. retina and optic nerve) causing vision loss or the efferent central nervous system ocular motor control networks (e.g. brainstem and cerebellum) causing nystagmus, abnormal eye tracking, and strabismus [2]. These paraneoplastic visual syndromes are a rare, but debilitating manifestation of malignancy.

Cancer-associated retinopathy (CAR) is a type of visual paraneoplastic syndrome that involves anti-retinal antibodies. Presenting symptoms typically involve bilateral visual disturbances such as a decline in visual acuity, photosensitivity, loss of color perception, night blindness, or scotomas [3]. Symptoms from CAR may present prior to the diagnosis of a malignancy, so early consideration of CAR can help aid in cancer detection. Unfortunately, current treatments for CAR have shown limited effectiveness in long-term visual outcomes. Since CAR is rare, it is helpful to highlight patient cases to share provider experiences with treating this disease. Furthermore, this case underscores the importance of considering paraneoplastic processes, which can in turn help recognize underlying malignancies.

## Case presentation

**Figure 1:**
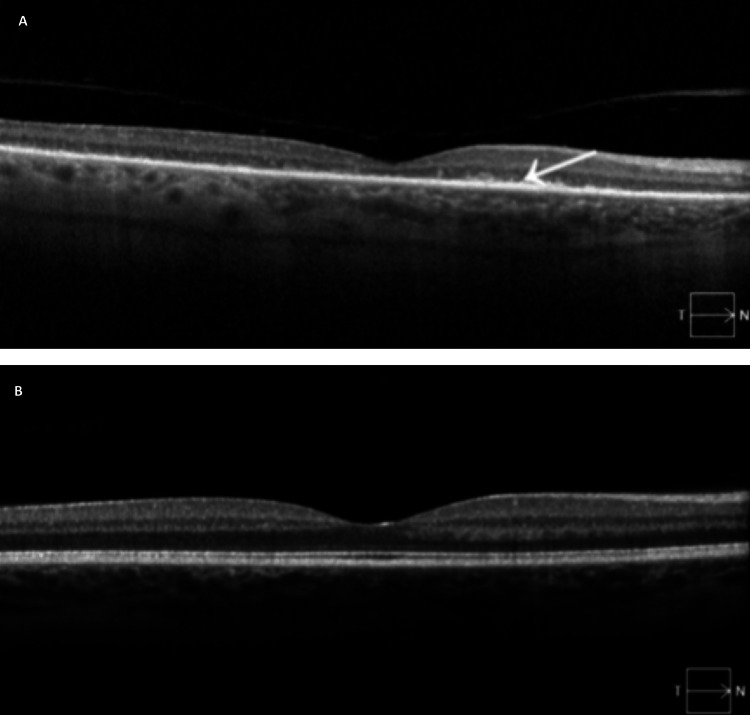
A) Case report patient’s right eye OCT macula with loss of the outer retinal layers (arrow) including the photoreceptor layer. B) Normal OCT macula of a healthy right eye, demonstrating well-preserved outer retinal layers.

A 72-year-old man with hypertension, hyperlipidemia, and a 40-pack-year smoking history presented to the neuro-ophthalmology clinic for progressive, bilateral vision loss. Over the span of six weeks, he noticed gradually worsening vision loss, where his visual acuity became limited to the extent that he was only able to detect hand motion movements in both eyes. His ophthalmologic exam revealed bilateral vitreous cells, but normal-appearing fundi without optic disc edema or chorioretinal changes. Optical coherence tomography (OCT) testing showed retinal nerve fiber layer atrophy of his bilateral optic nerves and OCT macula showed a near absence of the outer retinal layers, including the photoreceptors (Figure 1).

**Figure 2:**
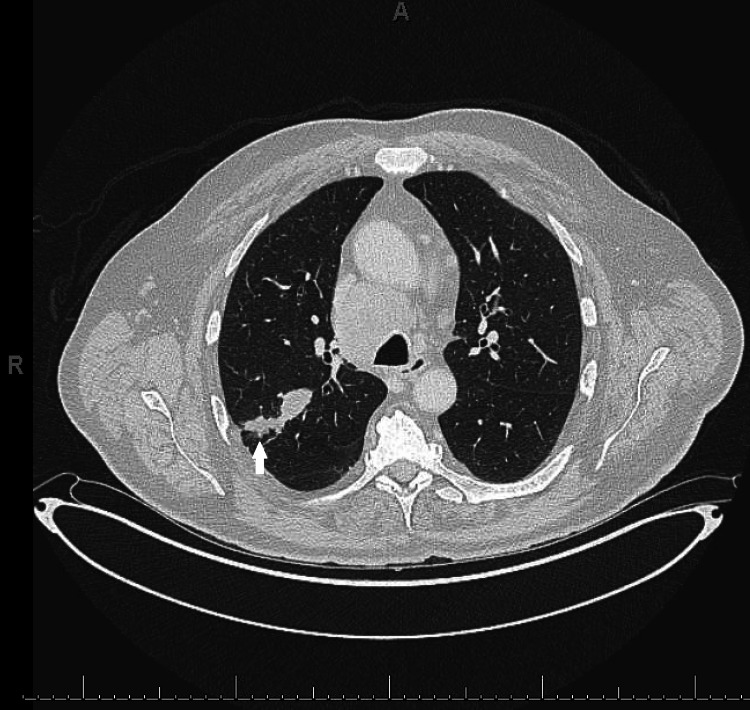
CT chest abdomen pelvis. Showing elongated, irregular, spiculated mass in the right upper lobe, measuring 5x1.6 cm in greatest axial dimension (white arrow).

**Figure 3:**
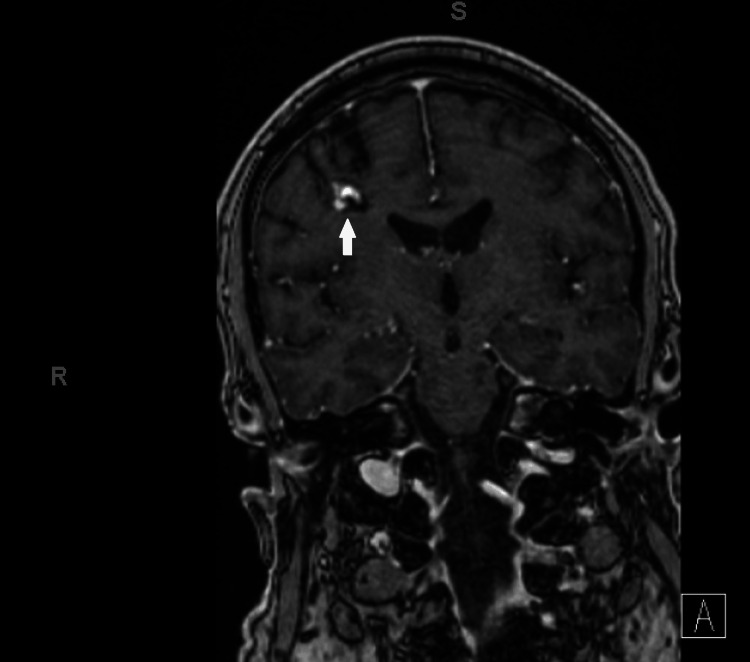
MRI brain coronal reconstruction view with IV contrast. Right frontal lobe cavernoma measuring 14mm (white arrow), no evidence of metastatic disease in the brain.

**Figure 4:**
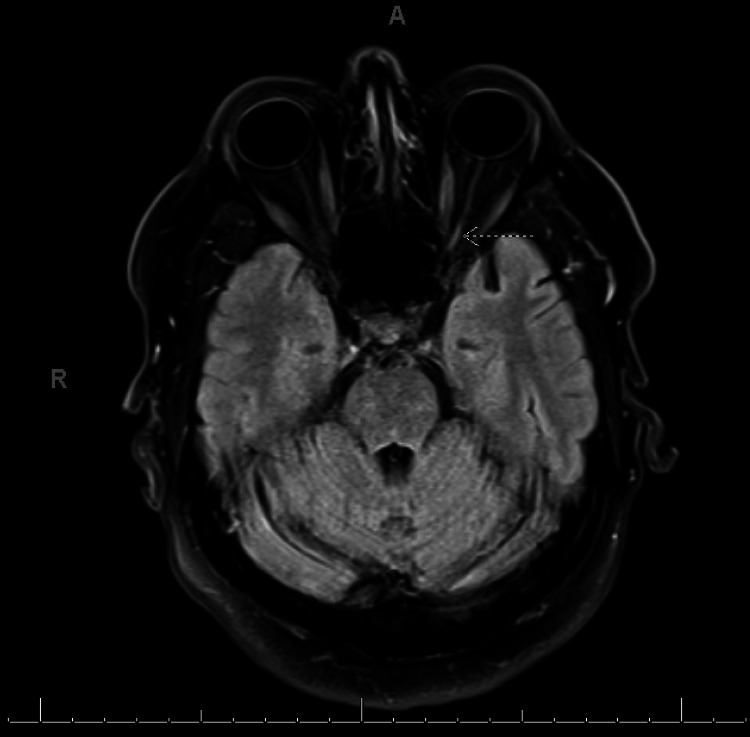
MRI orbit axial T2-weighted with fluid attenuated inversion recovery. Shows left greater than right optic nerve atrophy with nonspecific, high signal in the posterior intraorbital segment and canicular segment of the left optic nerve (white, dotted arrow).

Due to concerns for a paraneoplastic process, he had a CT chest, abdomen, and pelvis that revealed an irregular spiculated mass (5x1.5cm) in the right lung upper lobe, with mediastinal and cervical lymphadenopathy (Figure 2). A brain (Figure 3) and orbit (Figure 4) MRI did not show intracranial metastases nor evidence of inflammatory or neoplastic processes directly involving the optic nerves but did demonstrate optic nerve atrophy as previously seen on OCT testing. His retinopathy was treated with high-dose prednisone (starting at 60 mg/day and titrated down weekly as tolerated) and intravenous immunoglobulin (IVIG, 400 mg/kg daily for 5 days). His vision stabilized but unfortunately did not improve. His vision loss was suspected to be due to CAR. Positive serum anti-recoverin antibodies (titer>1: 1,800) secured the diagnosis of CAR. Of note, he was also screened for serum collapsin response mediator protein 5 (CRMP-5) antibodies for paraneoplastic optic neuropathy which were negative.

**Figure 5:**
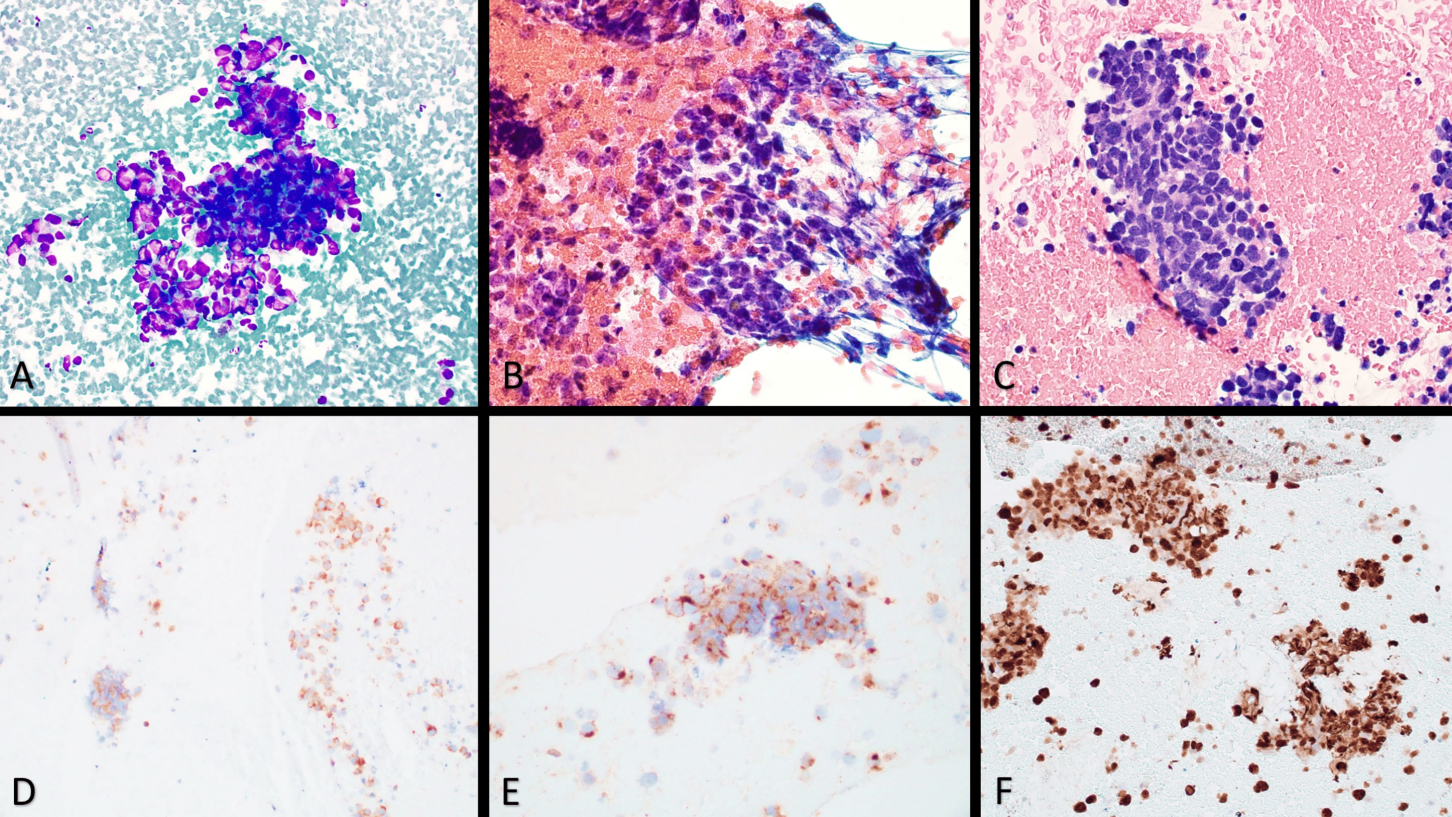
Histopathology images. A) Cohesive clusters of tumor cells with high nuclear to cytoplasmic ratios and moulding. Diff-Quik stain at 400X magnification. B) Clusters of tumor cells with salt and pepper chromatin and nuclear fragility. Papanicolaou stain at 600X magnification. C) Small cells with scant cytoplasm, hyperchromatic nuclei, moulding, and apoptotic debris. Hematoxylin & eosin stain at 400X magnification. D) Tumor cells expressing neuroendocrine marker synaptophysin. Synaptophysin immunohistochemistry at 200X magnification. E) Tumor cells expressing neuroendocrine marker chromogranin. Chromogranin immunohistochemistry at 400x magnification. F) High Ki-67 proliferation index of approximately 90%. Ki-67 immunohistochemistry at 200x magnification.

During his initial work-up and treatment for CAR, he underwent a mediastinal lymph node biopsy (Figure 5), which showed clusters of tumor cells that were diffusely positive for synaptophysin, chromogranin, and CD56, with a Ki-67 proliferation index of 90%. Overall, these results were consistent with a metastatic, poorly differentiated small cell lung cancer (SCLC). He was treated with carboplatin, etoposide, and atezolizumab (starting with cycle two, due to administrative and financial barriers to starting inpatient immunotherapy with cycle one) with a dramatic radiographic response after his second cycle. He completed four cycles of this therapy and was then advanced to maintenance atezolizumab. Although he had a very good response to systemic therapy, his vision showed only minimal recovery.

Eventually, he had a progression of disease while on maintenance atezolizumab and was switched to lurbinectedin. Initially, there was a tumor response to lurbinectedin, but on progression, his therapy was changed to topotecan, which had no systemic response. Finally, he began ipilimumab and nivolumab, but his course was complicated by hospitalization for acute urinary retention due to a Foley catheter malfunction followed by hypoxic respiratory failure due to a large loculated left-sided pleural effusion. He underwent two thoracentesis, which showed evidence of malignant, small-cell carcinoma in the pleural fluid. His pleural effusion quickly re-accumulated and after goals of care conversations, he chose to pursue comfort-based care. He died approximately 14 months after his initial SCLC diagnosis.

## Discussion

CAR is a rare type of paraneoplastic syndrome that is most frequently associated with malignancies such as SCLC, breast cancer, and gynecologic cancers [4]. However, CAR can be associated with a wide range of malignancies and case reports have described patients with other types of underlying cancers such as urothelial carcinoma, prostate cancer, and thymoma [5-7]. In more than half of these cases, visual symptoms preceded a cancer diagnosis, though there are several instances of CAR developing later, sometimes even years after an established malignancy. For example, one case report described a patient who was diagnosed with breast cancer and achieved complete remission but was later diagnosed with CAR 10 years later with no new malignancy detected on follow-up [8].

For this patient, other diagnostic considerations for his vision loss presentation included bilateral optic neuritis, primary central nervous system neoplasms compressing the chiasm (e.g. pituitary adenoma), or secondary metastasis to the optic nerves (e.g. lymphoma, colon carcinoma, etc.), paraneoplastic retinopathy from melanoma termed melanoma-associated retinopathy, autoimmune retinopathy, or retinitis pigmentosa. The patient’s relatively acute, bilateral visual decline, and profound outer retinal layer atrophy on OCT, with the lack of dermatologic or other systemic findings, placed paraneoplastic retinopathy at the top of the differential diagnosis. Other case reports have described heterogeneous presentations of CAR, though frequently noted findings such as visual field defects, ring scotoma, outer retinal atrophy, and diminished electroretinography may help clinicians consider CAR as part of their differential [5,9].

CAR involves anti-retinal antibodies, which can target a variety of onconeural antigens such as alpha-enolase, glyceraldehyde 3-phosphate dehydrogenase (GAPDH), carbonic anhydrase II, heat shock cognate protein 70 (Hsc70), and retinal S-antigen [10, 11]. While there have been a few case reports of seronegative CAR, most cases involve anti-retinal antibodies [11, 12]. Although anti-recoverin has low sensitivity, it does have high specificity (98%) for lung cancer [13]. It is believed that CAR develops as an immunogenic response to retinal epitopes on tumor cells, which lead to auto-antibodies that cross-react with tumor and retinal tissue. Anti-recoverin antibodies in particular are directed against a calcium-binding protein on retinal photoreceptor cells that regulate phosphorylation of rhodopsin [14]. Timely treatment of CAR with immunosuppression, such as steroids and IVIG, may improve vision and reduce the risk of permanent vision loss. However, visual prognosis tends to be poor, with immunosuppressive interventions showing mixed to limited responses [7,15].

It is unclear why visual acuity often does not improve, though we suspect that there is a component of irreversible retinal photoreceptor damage and cellular apoptosis that occurs within several weeks of initial vision symptoms. Visual changes are not necessarily correlated with the treatment response of the underlying cancer, and most patients with CAR will experience progressive visual loss or blindness eventually [16]. Some case studies have described a temporal improvement or slowing down of visual decline, but most reports have commented on an eventual worsening visual prognosis [17]. In addition to steroids and IVIG, other treatment strategies for CAR management have been considered such as early initiation of immunotherapy, plasmapheresis, and rituximab. Plasmapheresis in particular is theorized to be helpful in removing anti-recoverin antibodies, which may pause an immune attack. However given the rare occurrence of CAR and therefore lack of randomized clinical trials, there is a paucity of evidence-based best practices for CAR management. There remains a need for further research to identify best practices and targeted therapies to prevent or mitigate visual symptoms from CAR. Because of the rare occurrence of CAR much of our understanding of this disease is through shared experiences with case reports. While there are unique aspects with each case, this case report shares similar themes found in the literature, including the presence of an underlying SCLC, difficulty obtaining visual improvements, and the potential to recognize paraneoplastic symptoms prior to obtaining a diagnosis for an underlying malignancy.

## Conclusions

CAR can precede the diagnosis of cancer and can be an opportunity for early diagnosis of malignancy. Although this patient did not have improvement in his vision, astute recognition of bilateral outer retinopathy as a solitary exam finding of a paraneoplastic process led to the early identification and subsequent treatment of SCLC.

## References

[1] Pelosof LC, Gerber DE (2010). Paraneoplastic syndromes: an approach to diagnosis and treatment. Mayo Clin Proc.

[2] Kannoth S (2012). Paraneoplastic neurologic syndrome: A practical approach. Ann Indian Acad Neurol.

[3] Chan J (2003). Paraneoplastic retinopathies and optic neuropathies. Surv Ophthalmol.

[4] Dutta Majumder P, Marchese A, Pichi F, Garg I, Agarwal A (2020). An update on autoimmune retinopathy. Indian J Ophthalmol.

[5] Naramala S, Ahmad J, Adapa S, Gavini F, Konala VM (2019). Case series of cancer-associated retinopathy (car). Cureus.

[6] Duncan C, Zamir E, Teh J, Joon DL, Liodakis P (2019). A blind spot in urology: Prostate cancer associated retinopathy. Urol Case Rep.

[7] Katsuta H, Okada M, Nakauchi T (2002). Cancer-associated retinopathy associated with invasive thymoma. Am J Ophthal.

[8] Igarashi N, Sawamura H, Kaburaki T, Aihara M (2019). Cancer-associated retinopathy developing after 10 years of complete breast cancer remission. Neuroophthalmology.

[9] Hoogewoud F, Butori P, Blanche P, Brézin AP (2018). Cancer-associated retinopathy preceding the diagnosis of cancer. BMC Ophthalmol.

[10] Sculier C, Bentea G, Ruelle L (2017). Autoimmune paraneoplastic syndromes associated to lung cancer: A systematic review of the literature: Part 5: Neurological auto-antibodies, discussion, flow chart, conclusions. Lung Cancer.

[11] Adamus G, Ren G, Weleber RG (2004). Autoantibodies against retinal proteins in paraneoplastic and autoimmune retinopathy. BMC Ophthalmol.

[12] Thomas M, Benfield J, Morales J (2023). Case report of seronegative cancer-associated retinopathy in a patient with small cell lung carcinoma. Case Rep Oncol.

[13] Bazhin AV, Savchenko MS, Shifrina ON (2004). Recoverin as a paraneoplastic antigen in lung cancer: the occurrence of anti-recoverin autoantibodies in sera and recoverin in tumors. Lung Cancer.

[14] Bentea G, Sculier C, Grigoriu B, Meert AP, Durieux V, Berghmans T, Sculier JP (2017). Autoimmune paraneoplastic syndromes associated to lung cancer: A systematic review of the literature: Part 3: Neurological paraneoplastic syndromes, involving the central nervous system. Lung Cancer.

[15] Khan N, Huang JJ, Foster CS (2006). Cancer associated retinopathy (CAR): An autoimmune-mediated paraneoplastic syndrome. Semin Ophthalmol.

[16] Damek DM (2005). Paraneoplastic retinopathy/optic neuropathy. Curr Treat Options Neurol.

[17] Brossard-Barbosa N, Dezard V, Margolin E (2023). Treatment of cancer-associated retinopathy: A systematic literature review. Ophthalmol Retina.

